# Perampanel Add-on to Standard Radiochemotherapy *in vivo* Promotes Neuroprotection in a Rodent F98 Glioma Model

**DOI:** 10.3389/fnins.2020.598266

**Published:** 2020-11-30

**Authors:** Falko Lange, Jens Hartung, Clara Liebelt, Julius Boisserée, Tobias Resch, Katrin Porath, Max Frederik Hörnschemeyer, Gesine Reichart, Tina Sellmann, Valentin Neubert, Stephan Kriesen, Guido Hildebrandt, Elisabeth Schültke, Rüdiger Köhling, Timo Kirschstein

**Affiliations:** ^1^Oscar-Langendorff-Institute of Physiology, Rostock University Medical Center, Rostock, Germany; ^2^Center for Transdisciplinary Neurosciences Rostock, University of Rostock, Rostock, Germany; ^3^Department of Radiotherapy and Radiation Oncology, Rostock University Medical Center, Rostock, Germany

**Keywords:** epilepsy, glioblastoma, glioma, perampanel, radiochemotherapy, glutamate, glutamate receptors

## Abstract

An abnormal glutamate signaling of glioblastoma may contribute to both tumor progression and the generation of glioma-associated epileptic seizures. We hypothesized that the AMPA receptor antagonist perampanel (PER) could attenuate tumor growth and epileptic events. F98 glioma cells, grown orthotopically in Fischer rats, were employed as a model of glioma to investigate the therapeutic efficiency of PER (15 mg/kg) as adjuvant to standard radiochemotherapy (RCT). The epileptiform phenotype was investigated by video-EEG analysis and field potential recordings. Effects on glioma progression were estimated by tumor size quantification, survival analysis and immunohistological staining. Our data revealed that orthotopically-growing F98 glioma promote an epileptiform phenotype in rats. RCT reduced the tumor size and prolonged the survival of the animals. The adjuvant administration of PER had no effect on tumor progression. The tumor-associated epileptic events were abolished by PER application or RCT respectively, to initial baseline levels. Remarkably, PER preserved the glutamatergic network activity on healthy peritumoral tissue in RCT-treated animals. F98 tumors are not only a robust model to investigate glioma progression, but also a viable model to simulate a glioma-associated epileptiform phenotype. Furthermore, our data indicate that PER acts as a potent anticonvulsant and may protect the tumor-surrounding tissue as adjuvant to RCT, but failed to attenuate tumor growth or promote animal survival.

## Introduction

Glioblastoma (WHO grade IV) is a devastating disease with a median survival of 15 months (Delgado-López and Corrales-García, 2016) and severely reduced quality of life (QOL) due to cognitive decline and neurological deficits. Moreover, symptomatic epilepsy is frequently reported in patients with primary brain tumors, and in many cases an epileptic seizure is the initial symptom of malignant brain tumors (van Breemen et al., 2007). Low-grade gliomas (WHO grade I-II) exhibit a seizure prevalence ranging from 70 to 90%. In high-grade gliomas (WHO grade III-IV), up to 62% of the patients suffer from tumor-associated epilepsy (Kerkhof et al., 2013). Different mechanisms have been proposed to play a role in the generation of glioma-induced seizures (Huberfeld and Vecht, 2016). One major pathological mechanism is an altered glutamate signaling of glioma cells and their microenvironment. Our current understanding involves the glioma-expressed branched-chain amino acid transaminase 1 (BCAT1) that transfers α-amino groups from branched-chain amino acids to α-ketoglutarate, thereby producing glutamate and the respective branched-chain α-ketoacid (Tönjes et al., 2013). Intracellular glutamate, in turn, is exchanged for cystine via the xCT antiporter, which is often over-expressed in glioma tissue (Ye et al., 1999; Chung et al., 2005; Savaskan et al., 2008). This situation may be exacerbated by the downregulation or mislocalization of EAAT2 (excitatory amino acid transporter 2) which clears the neurotransmitter from the extracellular space (Ye et al., 1999; de Groot et al., 2005). It is assumed that these mechanisms contribute substantially to an elevated peritumoral glutamate level up to 100-times compared to unaffected brain tissue (Roslin et al., 2003; Marcus et al., 2010). High glutamate levels may promote proliferation and migration of glioma cells in an autocrine manner (Ishiuchi et al., 2007; Lyons et al., 2007) and also cause hyperexcitation of the surrounding neuronal tissue that eventually results in Ca^2+^-induced excitotoxicity of neurons (Noch and Khalili, 2009). Recently, this glioma model of glutamate interaction was expanded by the description of neurogliomal synapses (Venkataramani et al., 2019; Venkatesh et al., 2019). Synaptic transmission is based on glutamate and postsynaptic α-amino-3-hydroxy-5-methyl-4- isoxazolepropionic acid (AMPA) receptor expression, with the subunit GluA2 (GluR2) crucially involved. The authors demonstrated that glutamatergic signaling via integration of glioma cells into neuronal circuits promotes tumor cell growth and invasiveness.

Based on those pathological findings, anticonvulsants addressing tumor-associated seizures and, at the same time, interfering with glioma progression could be promising therapeutic candidates. Previous data suggested that the non-competitive AMPA receptor antagonist perampanel (PER) (Hanada et al., 2011) had antitumoral effects *in vitro* – unlike levetiracetam, valproate or carbamazepine (Lange et al., 2019), and attenuated patient-derived xenograft tumor growth in mice (Venkataramani et al., 2019). Furthermore, we have shown that systemic monotherapy of low-dose PER inhibited epileptiform discharges in organotypic brain slices of glioma (Mayer et al., 2019). Intriguingly, PER has been ascribed a neuroprotective effect in neurodegeneration occurring after pilocarpine-induced status epilepticus (Wu et al., 2017) and ischemia (Nakajima et al., 2018; Mazzocchetti et al., 2020).

Therefore, we established a rodent glioma model in order to evaluate the neuroprotective potential of PER as adjuvant treatment to standard radiochemotherapy (RCT). We demonstrated that orthotopic implantation of F98 glioma cells into Fischer 344 rats produced a valuable model to mimic orthotopic glioma progression in the rat neocortex with reduced survival and glioma-associated epilepsy. Our most important finding was that combined RCT and PER preserved physiological synaptic activity in the peritumoral tissue, while both RCT and PER alone were ineffective in this respect. These data suggest that PER promotes neuroprotection by reducing putatively detrimental glutamatergic effects within the peritumoral microenvironment.

## Materials and Methods

### Cell Culture

The rat F98 glioma cell line was obtained from the American Type Culture Collection (ATCC). F98 cells were cultured in Dulbecco’s modified eagle medium (DMEM)/F-12 (Merck, Darmstadt, Germany), supplemented with 10% fetal calf serum (FCS, Merck), and grown at 37°C in a 5% CO_2_ humidified atmosphere. In regular intervals, cell culture supernatants were tested for mycoplasma contamination employing MycoAlert Mycoplasma Detection Kit (Lonza, Basel, Switzerland).

### Animal Tumor Model and Stereotactic Glioma Implantations

F98 cells were implanted unilaterally into the sensomotor neocortex of Fischer 344 rats (Charles River, Sulzfeld, Germany) via stereotactic surgery to imitate human brain cancer (Mathieu et al., 2007). The experimental protocol of Mathieu et al. (2007) was modified with respect to the number of injected glioma cells, implantation volume, and position to fit with own studies in Wistar rats (Mayer et al., 2019). All procedures were conducted according to national and international guidelines on the ethical use of animals (European Council Directive 86/609/EEC, approval of local authority LALLF M-V/TSD/7221.3-1-020/20). All efforts were made to minimize animal suffering and to reduce the number of animals used. The animals were housed under environmentally controlled conditions (12 h light/dark cycles, lights switched on from 6 a.m. to 6 p.m., and 40–60% relative humidity). For the exploratory study a total of 105 Fischer 344 rats were included.

For the stereotactic glioma implantation, Fischer 344 rats (9–12 weeks old) were anesthetized with ketamine (100 mg/kg i.p.) and xylazine (10 mg/kg i.p.) and the head was fixed in a stereotactic frame (Narishige, Tokyo, Japan). Following a scalp incision, the skull was freed from extracranial muscles and a hole of 0.7 mm diameter was manually drilled into the skull in left parasagittal position (relative to bregma: 1.8 mm posterior, 2.5 mm left, 2 mm deep; positions are illustrated in Supplementary Figure 1A). Trypsinized F98 cells from a subconfluently growing culture were prepared for injection with a concentration of 10^4^ cells/μl phosphate-buffered saline (PBS). The glioma cell suspension was injected at a rate of 1 μl every 2 min (total of 10 μl, equivalent to 10^5^ cells) using a Hamilton syringe (Model 701 N SYR; Hamilton, Reno, NV, United States). After completing the injection, the drill hole was covered with Heliobond^®^, and the scalp was sutured. Sham-operated animals underwent the same procedure with 10 μl PBS instead of the cell suspension.

One week after glioma cell injection, Fischer 344 rats were randomly divided into four groups and treatment with PER only, RCT only or combined RCT/PER was started (see Supplementary Figure 1B for overview of experimental treatment protocol). Regardless of RCT or not, PER (15 mg/kg bw/day; Eisai Inc., Tokyo, Japan; formulated 1:1 in DMSO: PEG300) was delivered at a rate of 10 μl/h via a subcutaneously implanted, pre-loaded mini-osmotic pumps (Model 2ML1, Alzet, Cupertino, CA, United States). Sham-operated and F98-bearing cohorts w/o therapy received mini-osmotic pumps loaded with the vehicle only. Temozolomide (30 mg/kg bw/day; Selleck Chemicals, Houston, TX, United States) was administrated via intraperitoneal injections in a daily routine for five consecutive days (days 7–11 post-surgery, Figure 4A). On the same days, irradiation (5 × 4 Gy, a total of 20 Gy) of the whole brain (Supplementary Figures 1C–E) was performed in low-dose anesthesia with ketamine (20 mg/kg i.p.) and xylazine (2 mg/kg i.p.).

### Analysis of Video-EEG Recordings

Ten F98 glioma-bearing Fischer 344 rats were monitored by video-EEG (4 untreated, 2 RCT-treated, 2 Per-treated, and 2 RCT/PER-treated). To this end, these animals additionally received single-channel bipolar EEG recording during glioma implantation as previously described (Bajorat et al., 2011). Both electrodes were placed epidurally above the cortex with respect to the manufacturer’s instructions (electrode 1: 7.0 mm posterior to bregma, 1.5 mm left to sagittal suture; electrode 2: 2.0 mm anterior to bregma, 1.5 mm right to sagittal suture; Supplementary Figure 1A). Continuous 24/7 video-EEG data (sample rate 500 Hz, low-pass filter 30 Hz) were recorded employing a telemetric system (ETA-F20; Dataquest A.R.T.4.2., Data Sciences International, St. Paul, MN, United States) in combination with a light/dark network camera equipped with an infrared filter (Axis 223M; Axis Communications, Lund, Sweden). At nighttime, a small lamp over each cage improved video quality. Epileptiform potentials and seizures were analyzed manually by screening the video-EEG.

The video-EEG registration was maintained throughout the remaining lifetime of the animals. According to pre-defined humane endpoints, animals were sacrificed prior to reaching a moribund stage. While we aimed to investigate epileptiform potentials and epileptic seizures during the course of the tumor disease with or without therapy, we observed some variation in disease progression leading to variable survival. In order to compare similar clinical states, we defined a pre-final period as the time prior to reaching the humane endpoints from day 9 to 2 (relative to sacrifice). To rule out epileptiform activity due to the surgical procedure and/or anesthesia, we analyzed the EEG 12 h post-surgery for 12 consecutive hours.

### Neocortical Slice Preparations

For electrophysiological recordings, Fischer 344 rats were deeply anesthetized by diethyl ether inhalation (Mallinckrodt Baker, Deventer, Netherlands) and decapitated. The brain was quickly removed and transferred into chilled and oxygenated (95% O_2_/5% CO_2_) dissection solution containing (in mmol/l) 87 NaCl, 25 NaHCO_3_, 2.5 KCl, 1.25 NaH_2_PO_4_, 0.5 CaCl_2_, 7 MgCl_2_, 10 D-glucose and 75 sucrose adjusted to pH 7.4 with an osmolarity of 326–328 mosmol/l H_2_O. Next, the cerebellum was removed and the brain was sectioned (400 μm coronal slices) using a vibratome (Integraslice 7550 MM, Campden Instruments Ltd., United Kingdom) in chilled and oxygenated artificial cerebrospinal fluid (aCSF), comprised of (in mmol/l) 124 NaCl, 26 NaHCO_3_, 3 KCl, 1.25 NaH_2_PO_4_, 2.5 CaCl_2_, 1.5 MgCl_2_, and 10 D-glucose adjusted to pH 7.4 with an osmolarity of 304–312 mosmol/l H_2_O. After preparation, slices were transferred into a submerged-type storage chamber for maintenance with oxygenated aCSF and kept for 1.5 h of equilibrium, before starting recordings.

### Field Potential Recordings

For electrophysiological recordings, slices were transferred into an interface chamber (BSC-HT, Harvard Apparatus, Holliston, MA, United States) maintained at 32°C (TC-10, npi electronic GmbH, Tamm, Germany) and superfused with aCSF (perfusion rate of 2–3 ml/min). Under visual control electrodes were placed 500–1,000 μm from glioma above neocortical layers II/III and field potentials were recorded from with conventional aCSF-filled glass micropipette electrodes (Ag/AgCl with a resistance of approx. 2–5 MΩ). The analog recording data were amplified, filtered at 1 kHz by an EXT-10-2F (npi electronic GmbH), and digitized using a Micro1401 analog-to-digital converter (Cambridge Electronic Design, Cambridge, United Kingdom) run by the Signal 2.16 software (Cambridge Electronic Design). To evoke spontaneous physiological network activity in the brain tissue, the slices were exposed to three different aCSF solutions: (i) aCSF with 0 mM MgCl_2_ and addition of 5 μM gabazine (Tocris, Bristol, United Kingdom), (ii) aCSF with 8 mM KCl, 0 mM MgCl_2_ and 5 μM gabazine, (iii) aCSF with 0 mM MgCl_2_, 5 μM gabazine, and 50 μM 4-aminopyridine (4-AP; Tocris). Spontaneous physiological activity was defined as deflections with an amplitude at least twice the background potential and was observed under all conditions [(i): 20.3 ± 3.1 min^–1^, (ii): 37.9 ± 5.3 min^–1^, and (iii): 11.8 ± 1.2 min^–1^]. We chose protocol (i), since this evoked a medium incidence of spontaneous physiological activity; results of the other two protocols are shown in Supplementary Figure 2. To analyze *ex vivo* effects of PER on glutamatergic network activity, the anticonvulsant alone or in combination with D-AP5 [D-(-)-2-Amino-5-phosphonopentanoic acid; Tocris] were added to the aCSF solution.

### Tumor Size Quantification and Immunohistological Analysis

For histological analysis, F98 glioma-bearing brains of Fischer 344 rats were fixed in 3.7% paraformaldehyde phosphate buffer overnight, then cryo-protected with 30% sucrose in PBS overnight and frozen. For the quantification of tumor volume, brains were cut into 30-μm slices. The high expression of NeuN was used to distinguish between tumor cells from the surrounding tissue (Wolf et al., 1996). To this purpose, NeuN expression was detected employing an anti-NeuN primary antibody (Abcam, Ab104225, Cambridge, United Kingdom) and an anti-Rabbit IgG (H + L) Cross-Adsorbed, Cyanine5 (Thermo Fisher Scientific, Karlsruhe, Germany) as the secondary antibody. Afterward, the slices were counterstained and mounted with ProLong Gold Antifade Reagent containing 4′,6-diamidino-2-phenylindole (DAPI; Life Technologies, Darmstadt, Germany). Fluorescence analysis was performed by using a laser-scanning microscope (Leica DMI 6000, Wetzlar, Germany) and Leica Application Suite (v. 2.0.0.13332) software. After detection of F98 cells in frontal slices, the tumor area of every 500 μm was estimated and tumor volume was quantified as described before (Mayer et al., 2019). Routine hematoxylin and eosin staining for determination of tumor establishment was performed on 30-μm sections using standard procedures.

Additionally, the expression of the AMPA receptor subunit GluA2 in the tumor area, the peritumoral tissue and the contralateral hemisphere was determined using anti-GluA2 antibody (Alomone labs; AGC-005; Jerusalem, Israel) as the primary antibody and anti-rabbit Cyanine5 as the secondary antibody. For each animal in 1–3 slices (depending on the size of the tumor) GluA2 expression was determined. GluA2 data are presented as relative immunofluorescence (IF): IF_ROI_/IF_contralateral_. This ratio was normalized to IF_peritumoral_/IF_contralateral_ of the mean of untreated animals.

### Statistical Analysis

Statistical analysis was performed with SigmaPlot 13.0. Experimental results are illustrated in box plots or given as mean ± standard error of the mean (SEM) for the indicated number of experiments. Mean group differences were tested using non-parametric analysis of variance employing the Kruskal–Wallis test followed by *post hoc* Dunn’s test for multiple comparisons and Mann–Whitney *U* test for single comparison of two groups. For the analysis of spike load a two-way ANOVA followed by Bonferroni *t*-test was used. A significance level of *p* < 0.05 was considered to be statistically significant.

## Results

### Orthotopic F98 Glioma Is a Valuable Model for the Human Disease

**FIGURE 1:**
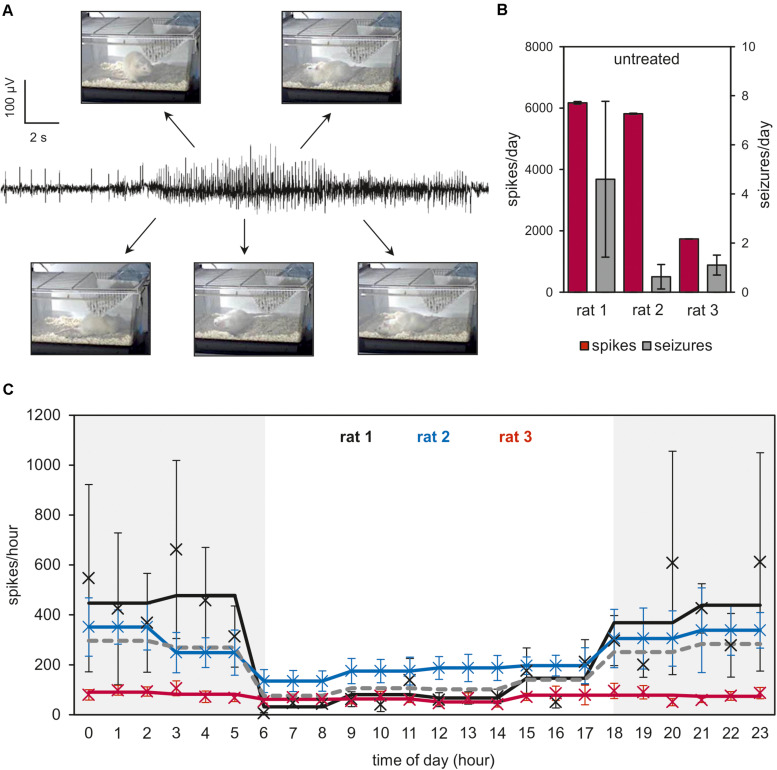
Video-EEG analysis of F98 glioma-bearing Fischer 344 rats. Single-channel electrodes were implanted epidurally to record an EEG trace of F98 glioma-bearing Fischer 344 rats for the whole time of the animal experiments. Rats were housed in individual cages with 12-h light/dark cycles (illumination: 6:00–18:00 h). **(A)** Sample trace of a representative seizure. Photos illustrate the association of field records with motor expressions during seizure. More details of ictal potentials are shown in Supplementary Figure 3. **(B)** The last 9–2 days (relative to sacrifice) in the life of the animals were analyzed and seizures were counted for each animal individually. **(C)** Number of spikes and distribution over time of day are shown as means ± SEM (5–9 data points per time of day). Means of 3-h-intervals are presented as lines. Gray-shaded areas indicate time of day without illumination in the animal housing facility. The dark gray dotted line represents mean of 3-h-intervals of all three animals.

First, we established a rat glioma model that shows both orthotopic glioma progression and tumor-associated symptomatic epilepsy. While *in vivo* implantation of F98 cells is a well-known glioma model (Kirschstein and Köhling, 2016), the epileptic phenotype has not yet been addressed. In prolonged video-EEG recordings, we could show that both epileptiform EEG potentials and motor seizures occurred during the pre-final clinical stage [from day 9 to 2 (relative to sacrifice); Figure 1A and Supplementary Figure 3]. Seizure frequency varied from 1–10 per day (Figure 1B), and the interictal spike load was also variable among the animals tested (Figure 1C). Obviously, spike load and seizure rates were poorly related in untreated animals. Intriguingly, there was a heterogeneous distribution of spikes between the light phase and the dark phase during the day. All analyzed animals showed a significantly higher spike load during the dark phase than during the light phase (*p* < 0.05, *U*-test; Figure 1C). Hence, F98 glioma-bearing animals develop symptomatic epilepsy.

**FIGURE 2:**
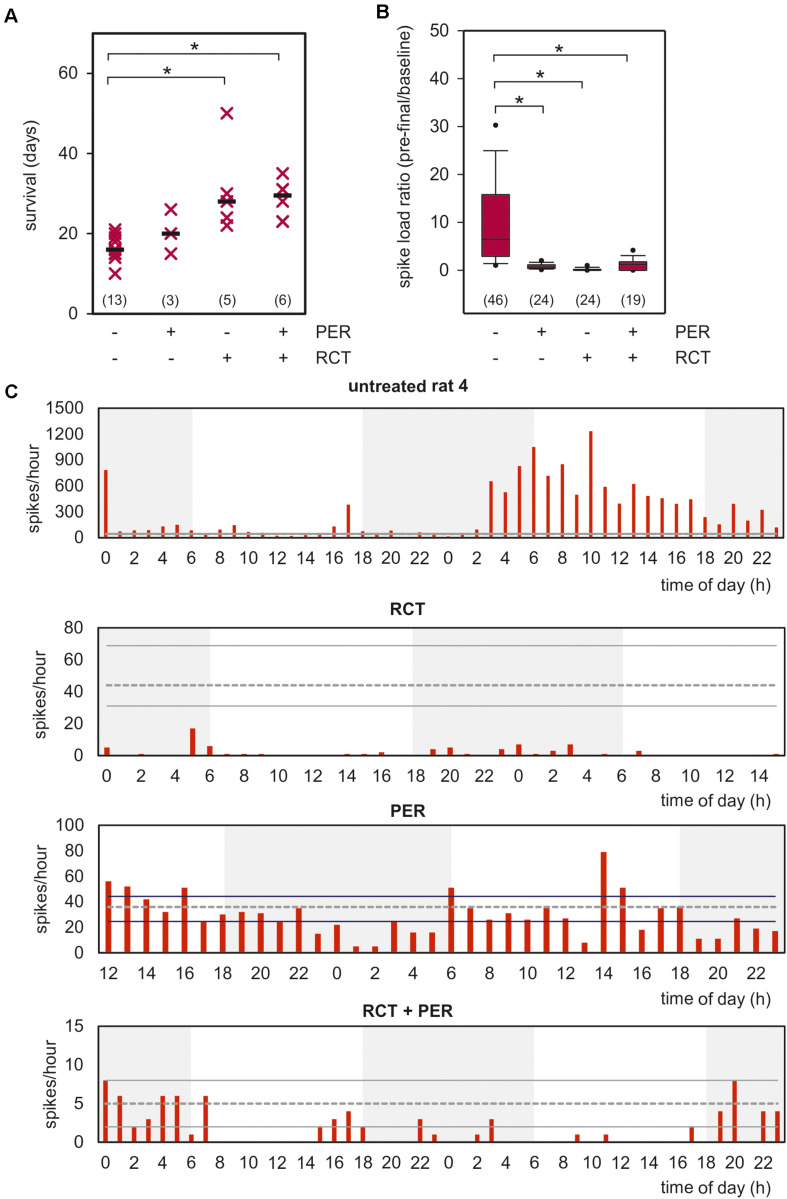
Impact of PER, RCT, and adjuvant PER treatment to RCT on clinical progression. **(A)** Rats with orthotopically-growing F98 glioma were sacrificed when endpoint criteria of the experiment design were reached and survival was estimated. Survival of each animals is illustrated as single point, median is shown as black bar (*n* = number of animals per group); *p < 0.05 [Kruskal–Wallis test followed by *post hoc* analysis (Dunn’s test)]. **(B)** Box plot shows video-EEG analysis of untreated animals versus RCT, PER, or RCT + PER cohorts. Data represent the number of analyzed hours in each cohort (data were obtained from 4 untreated, 2 RCT-treated, 2 PER-treated, and 2 RCT + PER-treated animals), *p < 0.05 [Kruskal–Wallis test followed by *post hoc* analysis (Dunn’s test)]. **(C)** Spike distribution over time of one animal from each investigated group is shown. Data range from 40–48 h of the last 3–4 days of the rats. Gray dotted line presents median of spikes of 12h-baseline as described in section “Materials and Methods,” whereas solid lines show upper and under quartiles.

A further prerequisite for a brain tumor model is reduced lifespan and limited response to standard tumor therapy. We tested the effect of standard RCT consisting of temozolomide (TMZ, 30 mg/kg) and radiotherapy (RT, 5 Gy) on five consecutive days (cumulative doses 150 mg/kg TMZ and 20 Gy, for treatment protocol see Supplementary Figure 1). As shown in Figure 2A, animals reached pre-defined humane endpoints 16.2 ± 0.8 days after glioma implantation, hence showed a drastically reduced survival. This finding is in line with previous published survival data (Mathieu et al., 2007). Although of little clinical significance, we treated three rats exclusively with PER (mean survival 20.3 ± 3.2 days), with no significant effects on survival in comparison to untreated animals. In marked contrast, combined whole-brain RT together with TMZ for five days significantly prolonged the survival to 30.8 ± 5.0 days (*p* < 0.05, one-way ANOVA on ranks followed by Dunn’s test). Adding PER (15 mg/kg) to this standard RCT could not prolong the survival (28.5 ± 2.4 days), but significantly reduced the inter-individual variance in our cohort (*p* < 0.05, *F*-test, Figure 2A). These findings demonstrate that PER for up to 50 days was well tolerated in F98 glioma-bearing animals showing a limited, but significant response to standard tumor therapy such as RCT.

Next, we asked whether tumor therapy affects the epileptic phenotype in our glioma model. To this end, we included a further glioma-bearing, but untreated animal in our EEG analysis to validate our previous results. Two animals received RCT alone or PER respectively, and two rats were given a combined therapy of RCT and PER. Since seizure rates differed substantially among the animals, we analyzed the spike load in the F98 model expressed as the ratio of spikes/h during the pre-final period relative to baseline values – as defined 12 h after tumor implantation (Figure 2B). These analyses revealed a reduction from 14.1 ± 4.1 in untreated animals to 0.8 ± 0.1 spikes/h in PER-treated animals which represents an attenuation down to 5.7% (*p* < 0.05, one-way ANOVA on ranks followed by Dunn’s test; Figure 2B). This anticonvulsive effect is in line with experiences from pilot studies of human glioma-associated epilepsy.

In RCT-treated rats spike load was reduced to 0.2 ± 0.1 spikes/h (1.4% of untreated animals; *p* < 0.05, one-way ANOVA on ranks followed by Dunn’s test; Figure 2B). The number of spikes during the pre-final stage was drastically reduced compared to post-surgery baseline (indicated by gray lines; Figure 2C). These findings indicate that RCT given in the second week after tumor implantation persistently suppressed the epileptic phenotype in later stages. It is important to note that this is in agreement with retrospective studies in humans also showing an anticonvulsive effect by TMZ and RT (Rudà et al., 2013; Koekkoek et al., 2015, 2016). Combined RCT-PER treatment also prevented the increase of spike load (1.2 ± 0.3; *p* < 0.05 versus untreated animals, one-way ANOVA on ranks followed by Dunn’s test; Figures 2B,C). The time courses of all four groups for the last 12-h interval are illustrated in Supplementary Figure 4. A two-way ANOVA followed by Bonferroni *t*-test revealed that no significant difference between time of day and treatment regime was found (Supplementary Figure 4). In line with this, seizures responded well to PER, regardless whether RCT was co-administered (Supplementary Figure 5).

**FIGURE 3:**
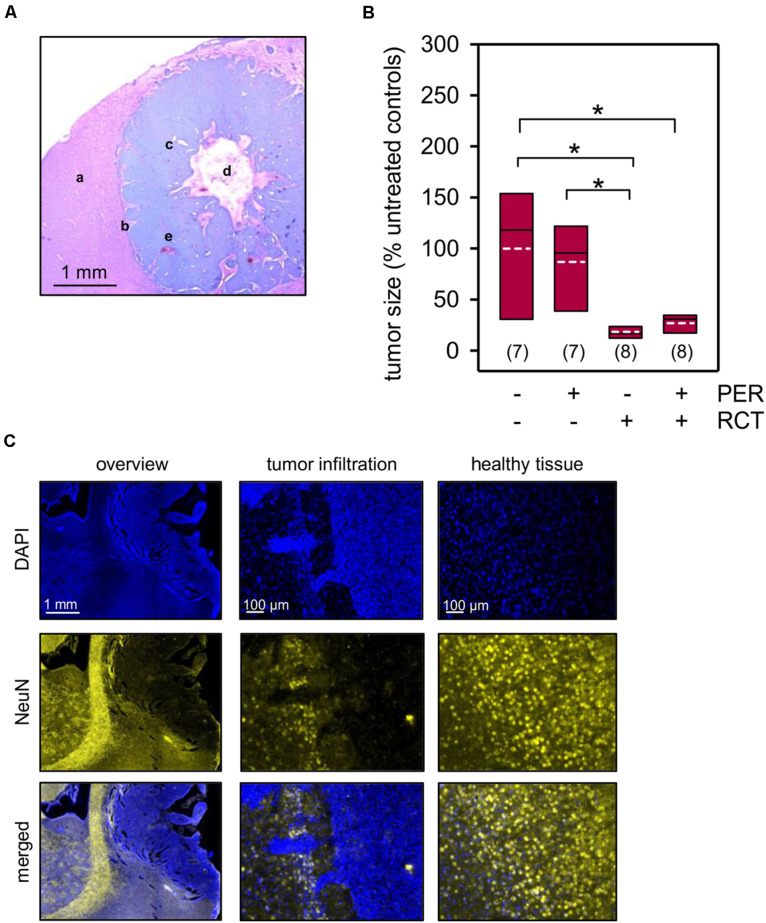
Tumor size quantification. Animals with orthotopically-growing F98 glioma were sacrificed after 2 weeks and brains were prepared for further histological and morphological analysis. **(A)** Hematoxylin and eosin staining confirms the presence of F98 glioma. The sample picture shows healthy brain tissue (a), tumor infiltration (b), glioma cells (c), central necrosis (d), and microvascular hyperplasia (e). **(B,C)** The tumor volume was quantified in a three-dimensionally manner by NeuN expression (yellow) of the healthy brain tissue, whereas F98 glioma presented only little or no expression of the marker protein. Nuclei were counterstained with DAPI (blue); white dotted line represents arithmetic mean, black solid line represents median; *n* = number of animals per group; *p < 0.05 [Kruskal–Wallis test followed by *post hoc* analysis (Dunn’s test)].

Prolonged survival following tumor therapy may predict a treatment-related reduction of tumor size. To test this directly, we analyzed the tumor size in histological sections 2 weeks after glioma implantation. The presence of the F98 tumor cells was verified by routine hematoxylin-eosin staining (Figure 3A), and the tumor size was three-dimensionally reconstructed using NeuN-based immunofluorescence micrographs (Figures 3B,C). While systemic administration of 15 mg/kg PER had only limited effects on F98 glioma tumor size (83.7 ± 14.7% of untreated animals), RCT significantly reduced the tumor volume to 17.1 ± 2.5% of untreated animals (*p* < 0.05, one-way ANOVA on ranks followed by Dunn’s test; Figure 3B). Adding PER, however, to this standard RCT had no further significant effect on glioma size (29.8 ± 5.3% of untreated animals). In summary, by implantation of F98 tumor cells into Fischer 344 rats we have established a valuable model of glioma-related mortality and morbidity including an epileptiform phenotype with response to standard RCT.

### Neuroprotective Effects of *in vivo* PER Add-on in the RCT-Treated Glioma Model

**FIGURE 4:**
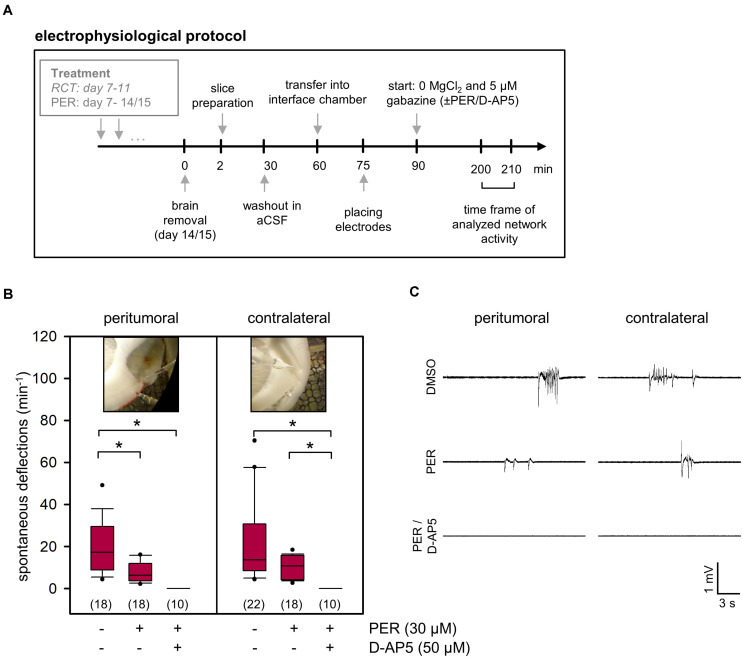
*Ex vivo* effects of PER on network activity in acute F98 glioma slices. **(A)** Illustration of the electrophysiological protocol for investigation of PER action on network activity. **(B)** 1 × 10^5^ F98 glioma cells were orthotopically injected into the neocortex of Fischer 344 rats. After 2 weeks the animals were sacrificed and brain slices were prepared for *ex vivo* application of vehicle control (DMSO), PER, NMDA receptor antagonist D-AP5, and combination thereof. Slices were challenged with aCSF with 0 μM Mg^2+^ and 5 μM gabazine. Data are represented in box plots, *n* = 10–22 measurements (slices were from a total of 16 rats with F98 glioma), *p < 0.05 (Kruskal–Wallis test with *post hoc* Dunn’s test). Photographs indicate peritumoral or contralateral position of microelectrodes. **(C)** Sample traces of field potential recording illustrate deflections that were used for quantification.

In order to evaluate PER effects on the physiological network activity within the peritumoral tissue, we induced spontaneous potential deflections in slices using specific recording solutions in pilot experiments (see section “Materials and Methods” and Supplementary Figure 2). Since we aimed to yield a medium incidence of spontaneous deflections, we chose a bath with 0 mM MgCl_2_ and 5 μM gabazine. Under these conditions, we obtained 20.3 ± 3.1 spontaneous deflections per minute in the peritumoral tissue from tumor-bearing, but otherwise untreated animals (Figures 4B,C). This network activity was significantly attenuated to 7.7 ± 1.1 events per minute by adding 30 μM PER to the bath (*p* < 0.05, one-way ANOVA on ranks followed by Dunn’s test), as expected from previous studies with rat and human glioma cell lines (Lange et al., 2019; Mayer et al., 2019). By further adding the NMDA receptor antagonist D-AP5 to the bath, the slices became electrically silent indicating that the network activity under our conditions was entirely glutamate-dependent. Similar results were obtained from the contralateral side suggesting that tumor infiltration in the corpus callosum or even parts of the contralateral hemisphere has occurred and affected glutamatergic network activity. Our results nicely demonstrate that PER, systemically applied prior to the brain preparation, had successfully been washed out before starting the *ex vivo* electrophysiological experiments.

**FIGURE 5:**
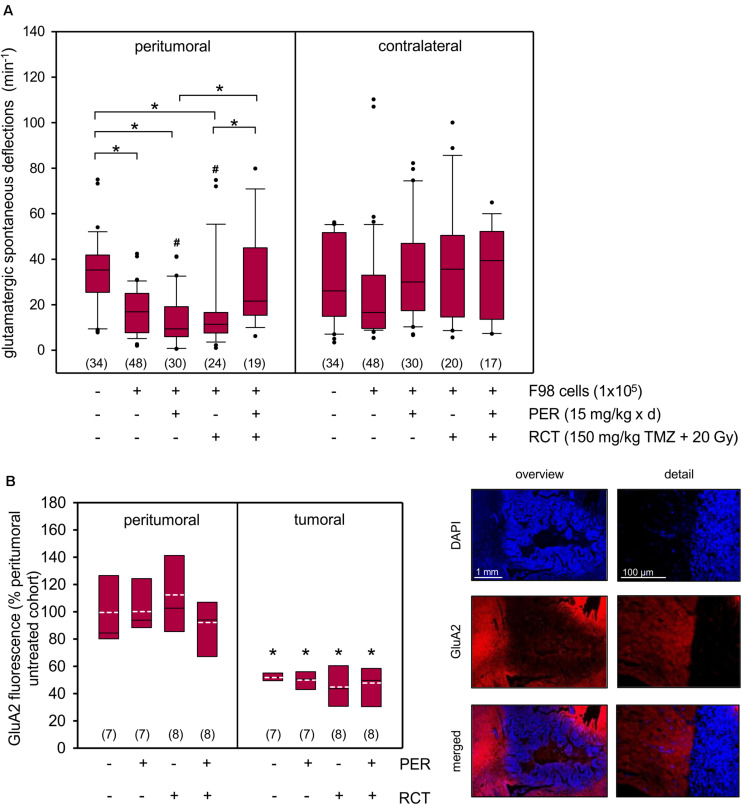
Synergistic impact of PER and RCT on glutamatergic network activity and GluA2 expression in acute F98 glioma slices. **(A)** One week after stereotactic injection of 1 × 10^5^ F98 cells, RCT with fractionated irradiation (5 × 4 Gy), concurrent administration of temozolomide (5 × 30 mg/kg bw) and an anticonvulsive treatment with PER (15 mg/kg bw/day) was started. After a total of 14–15 days animals were sacrificed and brain slices were prepared for electrophysiological analysis. The slices were exposed to aCSF 0 mM Mg^2+^ and 5 μM gabazine and field potential recordings were performed in surrounding tissue; *n* = 17–48 number of measurements (brain slices were from a total of 35 rats); *p < 0.05 (Kruskal–Wallis test followed by *post hoc* analysis (Dunn’s test); # < 0.05 versus contralateral equivalent (*U* test). **(B)** AMPA receptor subunit GluA2 expression was determined in the tumor area and peritumoral tissue of the ipsilateral hemisphere. Glu2A (red) immunofluorescence was normalized to peritumoral immunofluorescence intensity of the vehicle controls without PER or RCT (see section “Materials and Methods”). Nuclei were counterstained with DAPI (blue); white dotted line represents arithmetic mean, black solid line represents median. Significant difference between peritumoral and glioma fluorescence for each group was determined (*n* = 7–8 animals per group; *p < 0.05; *U* test).

Since we aimed to study neuroprotective effects of PER add-on to standard RCT in glioma *in vivo*, we provided the animals with subcutaneously localized osmotic pumps for continuous systemic PER treatment starting in parallel to the standard RCT. PER administration was continued until brain preparation on day 14–15 following tumor implantation, but was washed out before electrophysiological experiments (for protocol see Figure 4A). Next, we quantified the physiological synaptic network activity in five different cohorts: sham-operated animals, untreated glioma-bearing animals as well as animals with glioma and tumor therapy (in three arms: PER, RCT, and RCT/PER). Two weeks after tumor implantation, the physiological glutamatergic network activity in the peritumoral tissue was significantly reduced compared to sham-operated rats (16.9 ± 1.5 deflections/min versus 33.9 ± 2.8 deflections/min, *p* < 0.05, one-way ANOVA on ranks followed by Dunn’s test; Figure 5A) indicating severe disruption of synaptic connectivity. When glioma-bearing animals were treated with PER alone (13.1 ± 2.0 deflections/min, *p* < 0.05 versus sham, one-way ANOVA on ranks followed by Dunn’s test) or RCT alone (17.4 ± 3.9 deflections/min, *p* < 0.05 versus sham, one-way ANOVA on ranks followed by Dunn’s test), the induced physiological network activity was indistinguishable from that obtained in untreated F98 animals. This effect was specific for the peritumoral tissue, since on the contralateral side no differences were observed. However, the combination of the anticonvulsant PER together with RCT rescued the physiological network activity to 30.3 ± 4.7 deflections/min (*p* < 0.05 versus PER and *p* < 0.05 versus RCT, one-way ANOVA on ranks followed by Dunn’s test; Figure 5A). These values were almost identical to those observed in sham-operated animals, hence indeed suggesting neuroprotective effects by combined *in vivo* PER and RCT treatment in the F98 glioma model. This finding is corroborated by the comparison of ipsilateral versus contralateral network activity for each experimental group. Of all groups, only monotherapy with PER or RCT (for both groups: *p* < 0.05 ipsilateral versus contralateral, *U* test) presented lower values in ipsilateral recordings than contralateral, whereas a combination of RCT with adjuvant PER increased ipsilateral to contralateral network activity (Figure 5A). Interestingly, no differences were found in the cohort of untreated gliomas (*p* = 0.097, *U* test).

The question was therefore, whether the low effect of PER on tumor growth was associated with the expression of AMPA receptors, which we know to promote disease progression. GluA2 was selected as surrogate marker for AMPA receptors, as expression of this subunit was found to be associated with excitotoxicity and tumor invasion (Ishiuchi et al., 2007; Wright and Vissel, 2012). Our data indicate that F98 glioma express GluA2 roughly 50% (47–55%) of peritumoral tissue and treatment with PER, RCT or combination thereof did not affect expression levels of the AMPA receptor subunit (Figure 5B). Contralateral expression of GluA2 did not differ from ipsilateral peritumoral brain tissue (data not shown).

## Discussion

In high grade glioma and glioblastoma, the preservation of health-related QOL is an important therapeutic endpoint. Neurocognitive impairments and especially seizures are common and an anticonvulsant treatment is often indicated. Our major finding was that adding the AMPA receptor antagonist PER to standard RCT preserved network activity significantly more efficiently than standard RCT or PER alone in the peritumoral tissue of F98 glioma-bearing rats. Preserving network activity implies intact neuronal structures and thus may be regarded as neuroprotection in a sense of preserved neural function. This could be of great interest since most patients are offered a therapy approach including a RT regime (Weller et al., 2017, 2019). Therefore, we hypothesize that a tumor volume reduction by RCT alone is not sufficient to significantly protect the surrounding neurons. It also needs a second mechanism such as attenuation of AMPA receptor-mediated neuronal excitation via PER to mediate neuroprotection. Samari et al. (2013) demonstrated that inhibition of Ca^2+^ influx via NMDA receptors of irradiated neurons protects the cells from apoptosis. Furthermore, blocking of NMDA receptors prevents irradiation-induced abnormal glutamate signaling and synaptic remodeling (Duman et al., 2018). In addition, low doses of irradiation of glioma surrounding healthy brain tissue may lead to an enhanced migration and infiltration of glioma (Wank et al., 2018), which is presumed for F98 cells (Desmarais et al., 2016). PER may antagonize this key feature of glioma by blocking AMPA receptor-mediated migration (Ishiuchi et al., 2007; Piao et al., 2009).

The results of this study are in line with previous reports showing that F98 glioma progression can be decelerated by irradiation and concurrent administration of temozolomide (Wicks et al., 2015; De Meulenaere et al., 2019). The novel question in the focus of this study was whether PER can further improve the beneficial outcome. *In vitro* studies of glioblastoma and neuroblastoma cells indicated growth inhibition by the anticonvulsant (Lange et al., 2019; Nozawa et al., 2019) and a pilot study employing 12 patients suffering from glioblastoma correlated survival with PER plasma level (Izumoto et al., 2018). Here, no additional effect of PER on tumor size and survival was determined. Remarkably, in immunodeficient mice oral application of a high dose of PER reduced tumor cell density (Venkataramani et al., 2019). In that paper, slow-growing human glioma cells were used consistent with our previous study showing enhanced responsitivity to PER in human low-passage glioma cells (Lange et al., 2019). RCT reduced tumor size more than fivefold in comparison to untreated controls and animals had a 50% prolonged survival. One may speculate that potential effects of PER were disguised by the high impact of RCT. This negative finding is consistent with previous reports showing that AMPA receptor antagonists may fail to prolong survival in combination with RCT (Grossman et al., 2010; Iwamoto et al., 2010). Likewise, F98 cells could also be resistant to AMPA receptor inhibition. Glioblastoma often feature AMPA tetramers of GluA1 and GluA4 subunits (Corsi et al., 2019). It has been documented that F98 express all AMPA receptor subunits but GluA1 (Savaskan et al., 2011); a subunit that is associated with cell migration and adhesion in glioblastoma. In AMPA tetramers containing GluA2, Ca^2+^ permeability depends on this subunit. Therefore, the GluA2 subunit was chosen as surrogate marker for AMPA receptors. This subunit is subjected to a glutamine/arginine (Q/R) site RNA editing. AMPA receptors with edited GluA2 subunit are Ca^2+^ impermeable which also applies for F98 cells (Savaskan et al., 2011). In contrast, underedited GluA2 subunits allow the influx of Ca^2+^ that eventually may promote an augmented excitotoxicity of tumor surrounding neurons (Ishiuchi et al., 2007). Our immunohistological analyses revealed that F98 gliomas express the AMPA receptor subunit GluA2 and this expression is unaffected by RCT or PER, respectively.

Furthermore, we asked if an adjuvant therapy of PER affects the glioma-associated epileptiform phenotype. Remarkably, a combination of RT and temozolomide alone attenuated interictal spike load to initial baseline levels. These data are in line with observations made in patients with low-grade glioma in which RCT may contribute to a better seizure control (Koekkoek et al., 2015). For the first time, we showed that PER acts in an anticonvulsive manner in a glioma-associated rodent model of epilepsy. That was somewhat expected, as in several pilot studies with small sample sizes of glioma patients with drug-resistant epilepsy, high response rates to PER with improved seizure control or even seizure-free conditions were achieved (Vecht et al., 2017; Dunn-Pirio et al., 2018; Izumoto et al., 2018; Maschio et al., 2018; Chonan et al., 2020). Given the strong impact of RCT on the epileptiform phenotype, it is not surprising that no additional inhibitory effects by adjuvant PER were observed in our experiments.

To conduct the *in vivo* experiments with respect to orthotopic glioma progression and onset of tumor-related seizures, a robust animal model was needed. F98 glioma in Fischer rats is a well-established glioma model (Belloli et al., 2013; Schültke et al., 2018; Wang et al., 2018), but to the best of our knowledge, no data about its tumor-associated epileptiform phenotype have been published so far (Kirschstein and Köhling, 2016). The results of our studies suggest that F98 glioma led to interictal epileptiform events (e.g., spikes and spike-waves), indicating a high susceptibility to develop seizures, which was also demonstrated by video-EEG analysis. In all tested rats, the untreated animals suffered from seizures, but the occurrence of seizures is distributed heterogeneously between the animals. We find interictal events to be a robust surrogate marker to indicate the severity of the epileptiform phenotype. Interictal events are known to arise during glioma progression and are a subclinical marker of epilepsy. A recently published study suggests that an increasing frequency of interictal events may be associated with progressive neurologic impairment in glioma (Montgomery et al., 2020). The *ex vivo* presence of an glioma-associated epileptic phenotype is consistent with previous reports on resected human tissue (Köhling et al., 2006) and animal glioma models based on human genetic alterations (Hatcher et al., 2020). Together, our data demonstrate that the F98/Fischer 344 rat model is a suitable tool to investigate glioma-associated epilepsy in preclinical studies.

In conclusion, orthotopic implantation of F98 cells into the neocortex of Fischer 344 rats is a robust model of glioma progression. We have shown for the first time that this glioma model exhibits also an epileptiform phenotype. Furthermore, our data support the important role of glutamate and AMPA receptors in the context of glioma and its microenvironment. The administration of PER adjuvant to standard RCT led to neuroprotection in healthy glioma-surrounding brain tissue. This is important given the fact that radiotherapy is a crucial component in the treatment algorithms of glioma patients. However, PER failed to attenuate tumor growth or promote animal survival when administered adjuvant to RCT, but abolished the epileptiform phenotype of the rats.

## Data Availability Statement

The original contributions presented in the study are included in the article/Supplementary Material, further inquiries can be directed to the corresponding author.

## Ethics Statement

The animal study was reviewed and approved by Landesam für Landwirtschaft, Lebensmittelsicherheit und Fischerei Mecklenburg-Vorpommern, Thierfelderstraße 18, 18059 Rostock.

## Author Contributions

TK, FL, and ES designed the study. JH, TR, TS, MH, GR, KP, FL, and TK performed the experiments. ES and SK performed the irradiation. CL, JB, and VN analyzed the video-EEG recordings. GH provided materials to perform the experiments. FL wrote the manuscript. TK, ES, and RK contributed to parts of the manuscript and critically reviewed the final version of the manuscript. All authors analyzed and reviewed data.

## Conflict of Interest

The authors declare that the research was conducted in the absence of any commercial or financial relationships that could be construed as a potential conflict of interest.

## References

[B1] Bajorat R. Wilde M. Sellmann T. Kirschstein T. Köhling R. (2011). Seizure frequency in pilocarpine-treated rats is independent of circadian rhythm. *Epilepsia* 52 e118–e122. 10.1111/j.1528-1167.2011.03200.x 21801169

[B2] Belloli S. Brioschi A. Politi L. S. Ronchetti F. Calderoni S. Raccagni I. (2013). characterization of biological features of a rat F98 GBM model: a PET-MRI study with [18F]FAZA and [18F]FDG. *Nucl. Med. Biol.* 40 831–840. 10.1016/j.nucmedbio.2013.05.004 23915802

[B3] Chonan M. Saito R. Kanamori M. Osawa S. Watanabe M. Suzuki H. (2020). Experience of low dose perampanel to add-on in glioma patients with levetiracetam-uncontrollable epilepsy. *Neurol. Med. Chir.* 60 37–44. 10.2176/nmc.oa.2018-2245 PMC6970066 31748440

[B4] Chung W. J. Lyons S. A. Nelson G. M. Hamza H. Gladson C. L. Gillespie G. Y. (2005). Inhibition of cystine uptake disrupts the growth of primary brain tumors. *J. Neurosci.* 25 7101–7110. 10.1523/JNEUROSCI.5258-04.2005 16079392 PMC2681064

[B5] Corsi L. Mescola A. Alessandrini A. (2019). Glutamate receptors and glioblastoma multiforme: an old “Route” for new perspectives. *Int. J. Mol. Sci.* 20:1796. 10.3390/ijms20071796 30978987 PMC6479730

[B6] de Groot J. F. Liu T. J. Fuller G. Yung W. K. A. (2005). The excitatory amino acid Transporter-2 induces apoptosis and decreases glioma growth in vitro and in vivo. *Cancer Res.* 65 1934–1940. 10.1158/0008-5472.CAN-04-3626 15753393

[B7] De Meulenaere V. Bonte E. Verhoeven J. Kalala Okito J. P. Pieters L. Vral A. (2019). Adjuvant therapeutic potential of Tonabersat in the standard treatment of glioblastoma: a preclinical F98 glioblastoma rat model study. *PLoS One* 14:e0224130. 10.1371/journal.pone.0224130 31634381 PMC6802836

[B8] Delgado-López P. D. Corrales-García E. M. (2016). Survival in glioblastoma: a review on the impact of treatment modalities. *Clin. Transl. Oncol.* 18 1062–1071. 10.1007/s12094-016-1497-x 26960561

[B9] Desmarais G. Charest G. Therriault H. Shi M. Fortin D. Bujold R. (2016). Infiltration of F98 glioma cells in fischer rat brain is temporary stimulated by radiation. *Int. J. Radiat. Biol.* 92 444–450. 10.1080/09553002.2016.1175682 27121902

[B10] Duman J. G. Dinh J. Zhou W. Cham H. Mavratsas V. C. Pavešković M. (2018). Memantine prevents acute radiation-induced toxicities at hippocampal excitatory synapses. *Neuro Oncol.* 20 655–665. 10.1093/neuonc/nox203 29112734 PMC5892158

[B11] Dunn-Pirio A. M. Woodring S. Lipp E. Herndon J. E. Healy P. Weant M. (2018). Adjunctive perampanel for glioma-associated epilepsy. *Epilepsy Behav. Case Rep.* 10 114–117. 10.1016/j.ebcr.2018.09.003 30377587 PMC6202665

[B12] Grossman S. A. Ye X. Piantadosi S. Desideri S. Nabors L. B. Rosenfeld M. (2010). Survival of patients with newly diagnosed glioblastoma treated with radiation and temozolomide in research studies in the United States. *Clin. Cancer Res.* 16 2443–2449. 10.1158/1078-0432.CCR-09-3106 20371685 PMC2861898

[B13] Hanada T. Hashizume Y. Tokuhara N. Takenaka O. Kohmura N. Ogasawara A. (2011). Perampanel: a novel, orally active, noncompetitive AMPA-receptor antagonist that reduces seizure activity in rodent models of epilepsy. *Epilepsia* 52 1331–1340. 10.1111/j.1528-1167.2011.03109.x 21635236

[B14] Hatcher A. Yu K. Meyer J. Aiba I. Deneen B. Noebels J. L. (2020). Pathogenesis of peritumoral hyperexcitability in an immunocompetent CRISPR-based glioblastoma model. *J. Clin. Invest.* 130 2286–2300. 10.1172/jci133316 32250339 PMC7190940

[B15] Huberfeld G. Vecht C. J. (2016). Seizures and gliomas–towards a single therapeutic approach. *Nat. Rev. Neurol.* 12 204–216. 10.1038/nrneurol.2016.26 26965673

[B16] Ishiuchi S. Yoshida Y. Sugawara K. Aihara M. Ohtani T. Watanabe T. (2007). Ca2+-permeable AMPA receptors regulate growth of human glioblastoma via Akt activation. *J. Neurosci.* 27 7987–8001. 10.1523/JNEUROSCI.2180-07.2007 17652589 PMC6672718

[B17] Iwamoto F. M. Kreisl T. N. Kim L. Duic J. P. Butman J. A. Albert P. S. (2010). Phase 2 trial of Talampanel, a glutamate receptor inhibitor, for adults with recurrent malignant gliomas. *Cancer* 116 1776–1782. 10.1002/cncr.24957 20143438 PMC2846997

[B18] Izumoto S. Miyauchi A. Tasaki T. Okuda T. Nakagawa N. Nakano N. (2018). Seizures and tumor progression in glioma patients with uncontrollable epilepsy treated with perampanel. *Anticancer Res.* 38 4361–4366. 10.21873/anticanres.12737 29970574

[B19] Kerkhof M. Dielemans J. C. M. van Breemen M. S. Zwinkels H. Walchenbach R. Taphoorn M. J. (2013). Effect of valproic acid on seizure control and on survival in patients with glioblastoma multiforme. *Neuro Oncol.* 15 961–967. 10.1093/neuonc/not057 23680820 PMC3688020

[B20] Kirschstein T. Köhling R. (2016). Animal models of tumour-associated epilepsy. *J. Neurosci. Methods* 260 109–117. 10.1016/j.jneumeth.2015.06.008 26092434

[B21] Koekkoek J. A. F. Dirven L. Heimans J. J. Postma T. J. Vos M. J. Reijneveld J. C. (2016). Seizure reduction is a prognostic marker in low-grade glioma patients treated with temozolomide. *J. Neurooncol.* 126 347–354. 10.1007/s11060-015-1975-y 26547911 PMC4718947

[B22] Koekkoek J. A. F. Kerkhof M. Dirven L. Heimans J. J. Reijneveld J. C. Taphoorn M. J. B. (2015). Seizure outcome after radiotherapy and chemotherapy in low-grade glioma patients: a systematic review. *Neuro Oncol.* 17 924–934. 10.1093/neuonc/nov032 25813469 PMC5654353

[B23] Köhling R. Senner V. Paulus W. Speckmann E. J. (2006). Epileptiform activity preferentially arises outside tumor invasion zone in glioma xenotransplants. *Neurobiol. Dis.* 22 64–75. 10.1016/j.nbd.2005.10.001 16309916

[B24] Lange F. Weßlau K. Porath K. Hörnschemeyer J. Bergner C. Krause B. J. (2019). AMPA receptor antagonist perampanel affects glioblastoma cell growth and glutamate release in vitro. *PLoS One* 14:e0211644. 10.1371/journal.pone.0211644 30716120 PMC6361447

[B25] Lyons S. A. Chung W. J. Weaver A. K. Ogunrinu T. Sontheimer H. (2007). Autocrine glutamate signaling promotes glioma cell invasion. *Cancer Res.* 67 9463–9471. 10.1158/0008-5472.CAN-07-2034 17909056 PMC2045073

[B26] Marcus H. J. Carpenter K. L. H. Price S. J. Hutchinson P. J. (2010). In vivo assessment of high-grade glioma biochemistry using microdialysis: a study of energy-related molecules, growth factors and cytokines. *J. Neurooncol.* 97 11–23. 10.1007/s11060-009-9990-5 19714445

[B27] Maschio M. Pauletto G. Zarabla A. Maialetti A. Ius T. Villani V. (2018). Perampanel in patients with brain tumour-related epilepsy in real-life clinical practice: a retrospective analysis. *Int. J. Neurosci.* 129 593–597. 10.1080/00207454.2018.1555160 30507318

[B28] Mathieu D. Lecomte R. Tsanaclis A. M. Larouche A. Fortin D. (2007). Standardization and detailed characterization of the syngeneic Fischer/F98 glioma model. *Can. J. Neurol. Sci.* 34 296–306. 10.1017/S0317167100006715 17803026

[B29] Mayer J. Kirschstein T. Resch T. Porath K. Krause B. J. Köhling R. (2019). Perampanel attenuates epileptiform phenotype in C6 glioma. *Neurosci. Lett.* 715:134629. 10.1016/j.neulet.2019.134629 31734290

[B30] Mazzocchetti P. Mancini A. Sciaccaluga M. Megaro A. Bellingacci L. Di Filippo M. (2020). Low doses of perampanel protect striatal and hippocampal neurons against in vitro ischemia by reversing the ischemia-induced alteration of ampa receptor subunit composition. *Neurobiol. Dis.* 140:104848. 10.1016/j.nbd.2020.104848 32222474

[B31] Montgomery M. K. Kim S. H. Dovas A. Zhao H. T. Goldberg A. R. Xu W. (2020). Glioma-induced alterations in neuronal activity and neurovascular coupling during disease progression. *Cell Rep.* 31:107500. 10.1016/j.celrep.2020.03.064 32294436 PMC7443283

[B32] Nakajima M. Suda S. Sowa K. Sakamoto Y. Nito C. Nishiyama Y. (2018). AMPA receptor antagonist perampanel ameliorates post-stroke functional and cognitive impairments. *Neuroscience* 386 256–264. 10.1016/j.neuroscience.2018.06.043 29981363

[B33] Noch E. Khalili K. (2009). Molecular mechanisms of necrosis in glioblastoma: the role of glutamate excitotoxicity. *Cancer Biol. Ther.* 8 1791–1797. 10.4161/cbt.8.19.9762 19770591 PMC4503249

[B34] Nozawa A. Ozeki M. Matsuoka M. Nakama M. Yasue S. Endo S. (2019). Perampanel inhibits neuroblastoma cell proliferation through down-regulation of AKT and ERK pathways. *Anticancer Res.* 39 3595–3599. 10.21873/anticanres.13506 31262884

[B35] Piao Y. Lu L. de Groot J. (2009). AMPA receptors promote perivascular glioma invasion via beta1 integrin-dependent adhesion to the extracellular matrix. *Neuro Oncol.* 11 260–273. 10.1215/15228517-2008-094 18957620 PMC2718970

[B36] Roslin M. Henriksson R. Bergström P. Ungerstedt U. Bergenheim A. T. (2003). Baseline levels of glucose metabolites, glutamate and glycerol in malignant glioma assessed by stereotactic microdialysis. *J. Neurooncol.* 61 151–160. 10.1023/a:1022106910017 12622454

[B37] Rudà R. Magliola U. Bertero L. Trevisan E. Bosa C. Mantovani C. (2013). Seizure control following radiotherapy in patients with diffuse gliomas: a retrospective study. *Neuro Oncol.* 15 1739–1749. 10.1093/neuonc/not109 23897633 PMC3829585

[B38] Samari N. De Saint-Georges L. Pani G. Baatout S. Leyns L. Benotmane M. A. (2013). Non-conventional apoptotic response to ionising radiation mediated by N-Methyl D-aspartate receptors in immature neuronal cells. *Int. J. Mol. Med.* 31 516–524. 10.3892/ijmm.2013.1245 23338045 PMC3597540

[B39] Savaskan N. E. Heckel A. Hahnen E. Engelhorn T. Doerfler A. Ganslandt O. (2008). Small interfering RNA-mediated XCT silencing in gliomas inhibits neurodegeneration and alleviates brain edema. *Nat. Med.* 14 629–632. 10.1038/nm1772 18469825

[B40] Savaskan N. E. Seufert S. Hauke J. Tränkle C. Eyüpoglu I. Y. Hahnen E. (2011). Dissection of mitogenic and neurodegenerative actions of cystine and glutamate in malignant gliomas. *Oncogene* 30 43–53. 10.1038/onc.2010.391 20802520

[B41] Schültke E. Bräuer-Krisch E. Blattmann H. Requardt H. Laissue J. A. Hildebrandt G. (2018). Survival of rats bearing advanced intracerebral F 98 tumors after glutathione depletion and microbeam radiation therapy: conclusions from a pilot project. *Radiat. Oncol.* 13:89. 10.1186/s13014-018-1038-1036 PMC5946497 29747666

[B42] Tönjes M. Barbus S. Park Y. J. Wang W. Schlotter M. Lindroth A. M. (2013). BCAT1 promotes cell proliferation through amino acid catabolism in gliomas carrying Wild-Type IDH1. *Nat. Med.* 19 901–908. 10.1038/nm.3217 23793099 PMC4916649

[B43] van Breemen M. S. M. Wilms E. B. Vecht C. J. (2007). Epilepsy in patients with brain tumours: epidemiology, mechanisms, and management. *Lancet Neurol.* 6 421–430. 10.1016/S1474-4422(07)70103-5 17434097

[B44] Vecht C. Duran-Peña A. Houillier C. Durand T. Capelle L. Huberfeld G. (2017). Seizure response to perampanel in drug-resistant epilepsy with gliomas: early observations. *J. Neurooncol.* 133 603–607. 10.1007/s11060-017-2473-1 28492978

[B45] Venkataramani V. Tanev D. I. Strahle C. Studier-Fischer A. Fankhauser L. Kessler T. (2019). Glutamatergic synaptic input to glioma cells drives brain tumour progression. *Nature* 573 532–538. 10.1038/s41586-019-1564-x 31534219

[B46] Venkatesh H. S. Morishita W. Geraghty A. C. Silverbush D. Gillespie S. M. Arzt A. (2019). Electrical and synaptic integration of glioma into neural circuits. *Nature* 573 539–545. 10.1038/s41586-019-1563-y 31534222 PMC7038898

[B47] Wang K. Ha T. Chen X. Li S. Ai L. Ma J. (2018). A combined diffusion tensor imaging and Ki-67 labeling index study for evaluating the extent of tumor infiltration using the F98 Rat Glioma model. *J. Neurooncol.* 137 259–268. 10.1007/s11060-017-2734-z 29294232

[B48] Wank M. Schilling D. Schmid T. E. Meyer B. Gempt J. Barz M. (2018). Human glioma migration and infiltration properties as a target for personalized radiation medicine. *Cancers* 10:456. 10.3390/cancers10110456 30463322 PMC6266328

[B49] Weller M. Le Rhun E. Preusser M. Tonn J. C. Roth P. (2019). How we treat glioblastoma. *ESMO Open.* 4(Suppl. 2):e000520. 10.1136/esmoopen-2019-000520 31297242 PMC6586206

[B50] Weller M. van den Bent M. Tonn J. C. Stupp R. Preusser M. Cohen-Jonathan-Moyal E. (2017). European association for neuro-oncology (EANO) guideline on the diagnosis and treatment of adult astrocytic and oligodendroglial gliomas. *Lancet Oncol.* 18 e315–e329. 10.1016/S1470-2045(17)30194-8 28483413

[B51] Wicks R. T. Azadi J. Mangraviti A. Zhang I. Hwang L. Joshi A. (2015). Local delivery of cancer-cell glycolytic inhibitors in high-grade glioma. *Neuro Oncol.* 17 70–80. 10.1093/neuonc/nou143 25053853 PMC4483048

[B52] Wolf H. K. Buslei R. Schmidt-Kastner R. Schmidt-Kastner P. K. Pietsch T. Wiestler O. D. (1996). NeuN: a useful neuronal marker for diagnostic histopathology. *J. Histochem. Cytochem.* 44 1167–1171. 10.1177/44.10.8813082 8813082

[B53] Wright A. L. Vissel B. (2012). The essential role of AMPA receptor GluR2 subunit RNA editing in the normal and diseased brain. *Front. Mol. Neurosci.* 5:34. 10.3389/fnmol.2012.00034 22514516 PMC3324117

[B54] Wu T. Ido K. Osada Y. Kotani S. Tamaoka A. Hanada T. (2017). The neuroprotective effect of perampanel in lithium-pilocarpine rat seizure model. *Epilepsy Res.* 137 152–158. 10.1016/j.eplepsyres.2017.06.002 28624183

[B55] Ye Z. C. Rothstein J. D. Sontheimer H. (1999). Compromised glutamate transport in human glioma cells: reduction-mislocalization of sodium-dependent glutamate transporters and enhanced activity of cystine-glutamate exchange. *J. Neurosci.* 19 10767–10777. 10.1523/jneurosci.19-24-10767.1999 10594060 PMC6784962

