# Supplementary material for: Perampanel Add-on to Standard Radiochemotherapy in vivo Promotes Neuroprotection in a Rodent F98 Glioma Model
Source: Front Neurosci. 2020 Nov 30;14:598266. doi: 10.3389/fnins.2020.598266 (PMC7734300; doi:10.3389/fnins.2020.598266)
Supplement: Supplementary file 1 [file Data_Sheet_1.pdf]

Supplementary Material

Experimental treatment protocol

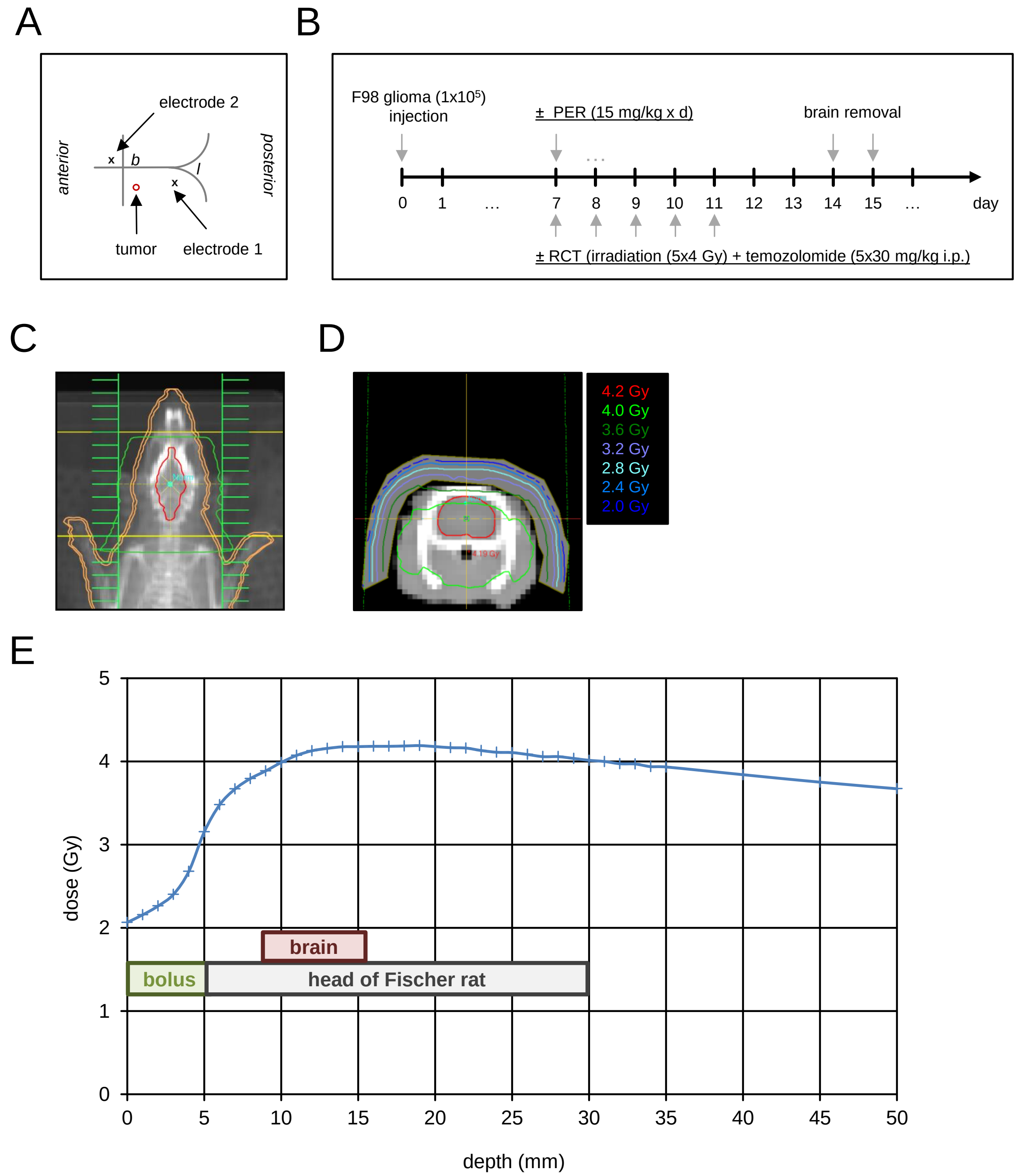

Supplementary Figure 1. Experimental treatment protocol. **(A)** stereotactic coordinates of tumor injection (1.8 mm posterior, 2.5 mm left), EEG electrode 1 (7.0 mm posterior, 1.5 mm left), and EEG electrode 2 (2.0 mm anterior, 1.5 mm right) in reference to bregma (b); l=lambda. **(B)** Illustration of the experimental approach for investigation of PER action in combination with a standard RCT protocol. **(C)** Beams eye view of the one field treatment plan for Fischer rat **(D)** Simulation of dose distribution. **(E)** Dosimetry calculation for irradiation of F98 glioma in Fischer 344 rats.

Network activity models of F98 glioma slices

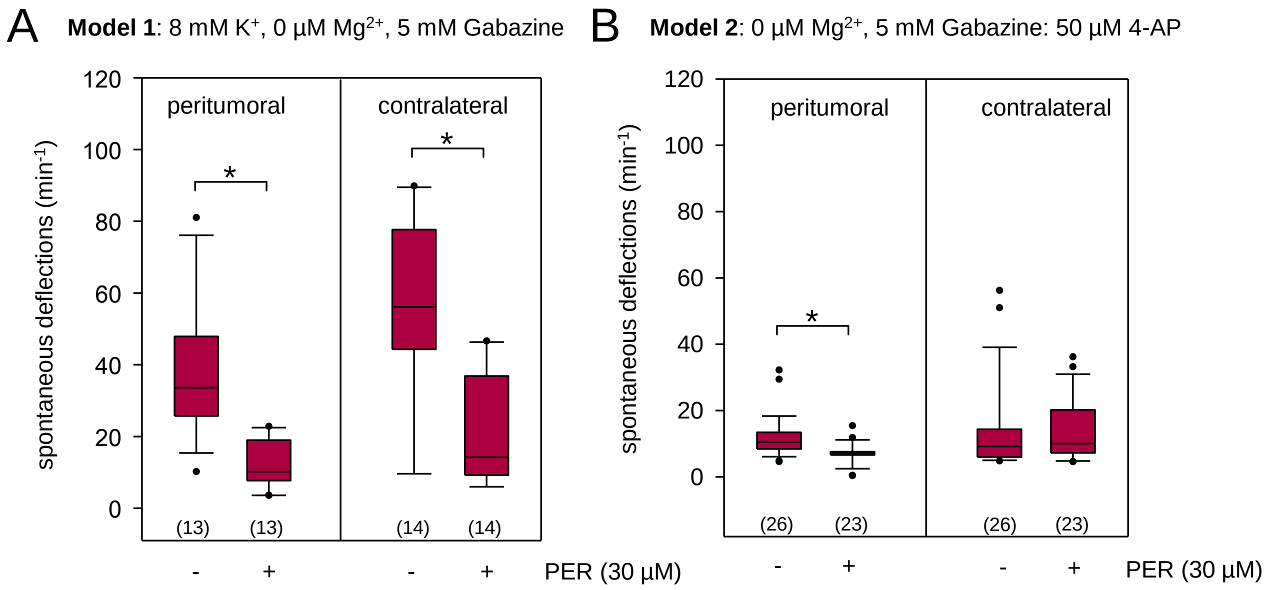

Supplementary Figure 2. Additional network activity models of F98-glioma brain slices. F98 glioma-bearing Fischer 344 rats were sacrificed two weeks after tumor implantation and acute brain slices were exposed two ex vivo models of network activity: **(A)** model 1: 8 mM K^+, 0 mM Mg^{2+}, and 5 µM gabazine; **(B)** model 2: 0 mM Mg^{2+}, 5 µM gabazine, and 50 µM 4-Aminopyridine. Furthermore, slices were challenged to PER (30 µM). Data are represented in box plots, n=13-26, *p<0.05 (Kruskal-Wallis test with post hoc Dunn's test).

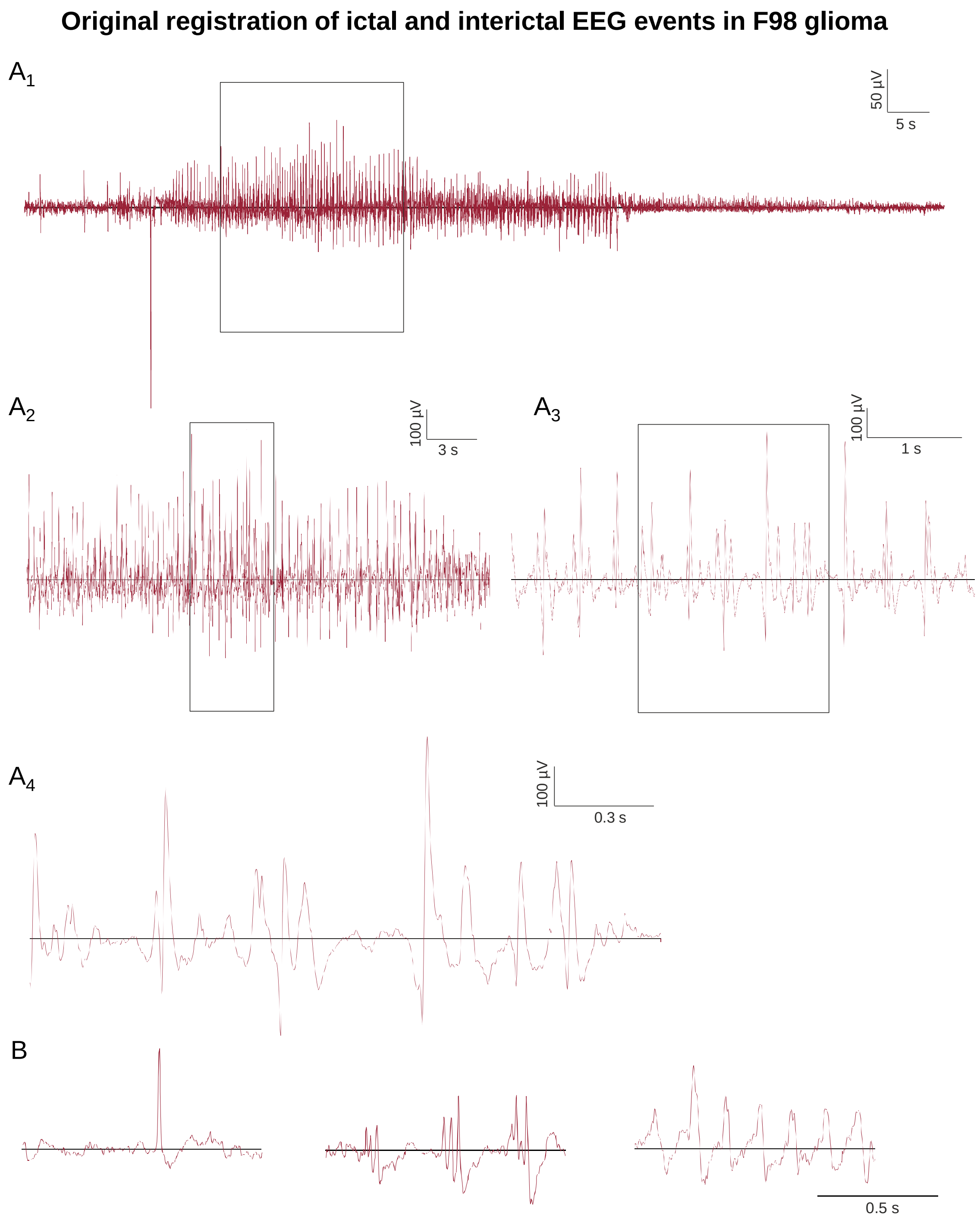

Supplementary Figure 3. Epileptiform potentials of video-EEG recordings in F98 glioma. Single-channel 24/7 video-EEG data were screened manually. Traces illustrate sample EEG potentials of **(A)** ictal (shown are more details of the seizure presented in Figure 1 and potentials in details) and **(B)** interictal discharges (left to right: spike, polyspike and spike-wave) in untreated Fischer 344 rats harboring F98 glioma

Spike load of all experimental groups with glioma

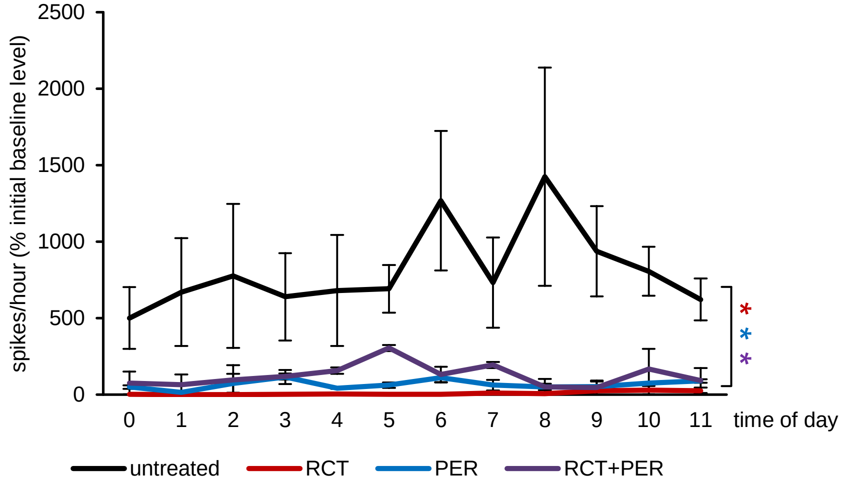

Supplementary Figure 4. Time course of interictal spike load of all four experimental groups with glioma. Data represent the mean±SEM of last 12 hours in each cohort as percent of initial baseline level after surgery. Data were obtained from 4 untreated, 2 RCT-treated, 2 PER-treated, and 2 RCT+PER-treated animals. *p<0.05 (two-way ANOVA followed by Bonferroni t-test). No significant difference between time of day and treatment regime was found.

Seizure occurrence of all experimental groups with glioma

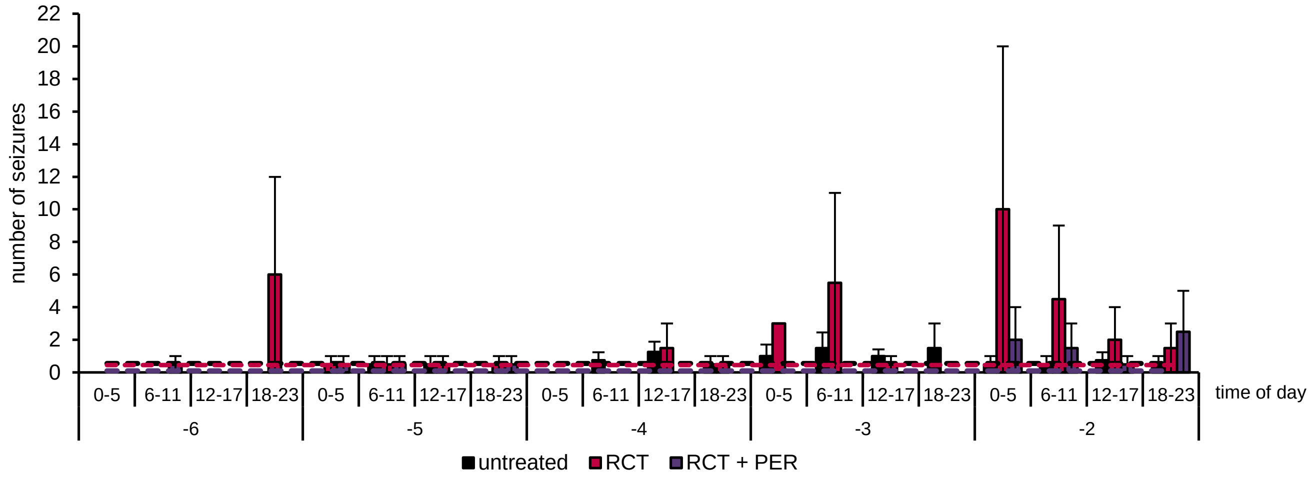

Supplementary Figure 5. Seizure occurrence of all four experimental groups with glioma. Data represent the mean of seizure on day -6 to -2 (relative to sacrifice). Dotted lines represent median of seizure of all five analyzed days. Please note that in PER-treated animals no seizures were detected.

Table 1: Survival (in days) of Fischer 344 rats after orthotopic injection of $1x10^5$ F98 glioma cells

no. of animals	untreated	RCT	PER	RCT+PER
1	19	30	15	31
2	18	50	26	31
3	20	22	20	23
4	16	24		35
5	16	28		28
6	15			23
7	16			
8	15			
9	21			
10	10			
11	16			
12	14			
13	15			
